# Radiotherapy-induced ferroptosis for cancer treatment

**DOI:** 10.3389/fmolb.2023.1216733

**Published:** 2023-06-14

**Authors:** Giovanni L. Beretta, Nadia Zaffaroni

**Affiliations:** Molecular Pharmacology Unit, Department of Experimental Oncology, Fondazione IRCCS Istituto Nazionale dei Tumori di Milano, Milan, Italy

**Keywords:** ferroptosis, radiotherapy, reactive oxygen species, nanomedicine, drug combinations, gene signatures

## Abstract

Ferroptosis is a regulated cell death mechanism controlled by iron, amino acid and reactive oxygen species metabolisms, which is very relevant for cancer therapy. Radiotherapy-induced ferroptosis is critical for tumor suppression and several preclinical studies have demonstrated that the combination of ionizing radiation with small molecules or nano-systems is effective in combating cancer growth and overcoming drug or ionizing radiation resistance. Here, we briefly overview the mechanisms of ferroptosis and the cross-talk existing between the cellular pathways activated by ferroptosis and those induced by radiotherapy. Lastly, we discuss the recently reported combinational studies involving radiotherapy, small molecules as well as nano-systems and report the recent findings achieved in this field for the treatment of tumors.

## 1 Introduction

The accidental cell death (ACD) and the regulated cell death (RCD) govern the cell fate (Tang et al., 2019). Necrosis is the best representative of ACD, which is a passive mechanism allowing plasma membrane rupture, cytoplasm release and inducing inflammation reaction. Conversely, RCD is an active and regulated cell suicide, which can involve or not inflammation reaction, playing crucial functions in tissue homeostasis and in the pathogenesis of several diseases (Galluzzi et al., 2018; Tang et al., 2019). So far, two RCD categories are reported: apoptotic and non-apoptotic. Necroptosis, pyroptosis, autophagy and ferroptosis belong to the non-apoptotic RCD and are classified according to different molecular, morphological, biochemical and functional features (Galluzzi et al., 2018). RCD pathways are implicated in physiologic processes regulating the development of multicellular organisms and represent defense mechanisms against cancer transformation/development as well as against pathogen infections (Galluzzi et al., 2018; Tang et al., 2019). As suggested by its name, ferroptosis is stimulated by the lipid peroxidation provoked by the iron accumulated into the cells. Critical for ferroptosis is the content of polyunsaturated-fatty-acids (PUFA) composing the cellular membrane. PUFA represent a toxic reservoir that in iron- and reactive oxygen species (ROS)-rich conditions are susceptible to peroxidation, leading to membrane damage and cell death (Stockwell et al., 2017).

The induction of ferroptosis is a new interesting strategy for fighting cancer (Lei et al., 2022). This strategy is based on the condition known as iron addiction, which is typical of cancer cells that need higher levels of iron in comparison with healthy cells (Friedmann Angeli et al., 2019). Iron addiction renders cancer cells more sensitive to iron and to iron-induced ROS production (Fenton reaction) than normal cells (Torti and Torti, 2019). Though ferroptosis induction proved efficacy in overcoming resistance to apoptosis developed by tumors exposed to anticancer therapy, tumor cells challenged by ferroptosis inducers can evolve defense mechanisms that counteract ferroptosis and in turn preserving cell vitality (Stockwell et al., 2017; Hassannia et al., 2019; Zheng and Conrad, 2020). This implies that drugs hitting cellular pathways involved in resistance to ferroptosis potentiate pharmacological interventions (Liang et al., 2019).

Besides chemotherapy, tumors are treated by radiotherapy as well. The exposure to ionizing radiation (IR) induces DNA damage leading to cell death (Delaney et al., 2005; Jaffray, 2012). Besides direct DNA damage, IR hits water molecules contained into the cells favoring their radiolysis and, together with the activation of specific enzymes, stimulate ROS production. ROS, including peroxides (H_2_O_2_, ROOH), free radicals (HO•, HO_2_•, R•, RO•, NO• and NO_2_•), singlet oxygen (^1^O_2_) and superoxide (O_2_−•), attack DNA, lipids, and proteins (Azzam et al., 2012; Reisz et al., 2014). DNA damages include nucleotide base damage, single strand breaks (SSBs), and double strand breaks (DSBs) (Baidoo et al., 2013). The cellular response to damaged DNA allows cell-cycle arrest, cellular senescence, and RCD. Although apoptosis is the most studied RCD induced by radiotherapy, other types of RCD are reported in radiotherapy-treated cells, including ferroptosis (Lang et al., 2019; Adjemian et al., 2020; Lei et al., 2020; Ye et al., 2020). Since for the treatment of tumors radiotherapy is administered in combination with chemotherapy, it is conceivable to study the radiotherapy and ferroptosis pathways as well as their cross-talk to set up combination strategies maximizing tumor response.

In this review we briefly overview the cellular pathways implicated in ferroptosis induced by radiotherapy and discuss the potential combination strategies with small molecules and nano-systems for enhancing radiotherapy antitumor activity.

## 2 General overview on ferroptosis

Compared to apoptosis, autophagy and necrosis, ferroptosis shows peculiar properties. Morphological alterations of mitochondria, including reduction in volume, increased density of the mitochondrial membranes as well as reduced mitochondrial cristae, characterize ferroptotic cells. Cells undergoing ferroptosis are rounded and floating with intact nuclei and uncondensed chromatin (Dixon et al., 2012; Stockwell et al., 2017). Conversely, the typical features of apoptosis (e.g., chromatin condensation and the production of apoptotic bodies) as well as that of autophagy (e.g., the formation of autophagosomes) are not reported in ferroptotic cells. Agents that inhibit apoptosis, autophagy and necroptosis are ineffective on ferroptosis induction. Therefore, the sensitivity to drugs that induce ferroptosis is maintained by cells deficient in apoptotic-related factors (e.g., BAX, BAK, MLKL, and RIPK1/3). On the contrary, antioxidants and iron chelators inhibit ferroptosis (Vanden Berghe et al., 2010; Lei et al., 2019).

**FIGURE 1:**
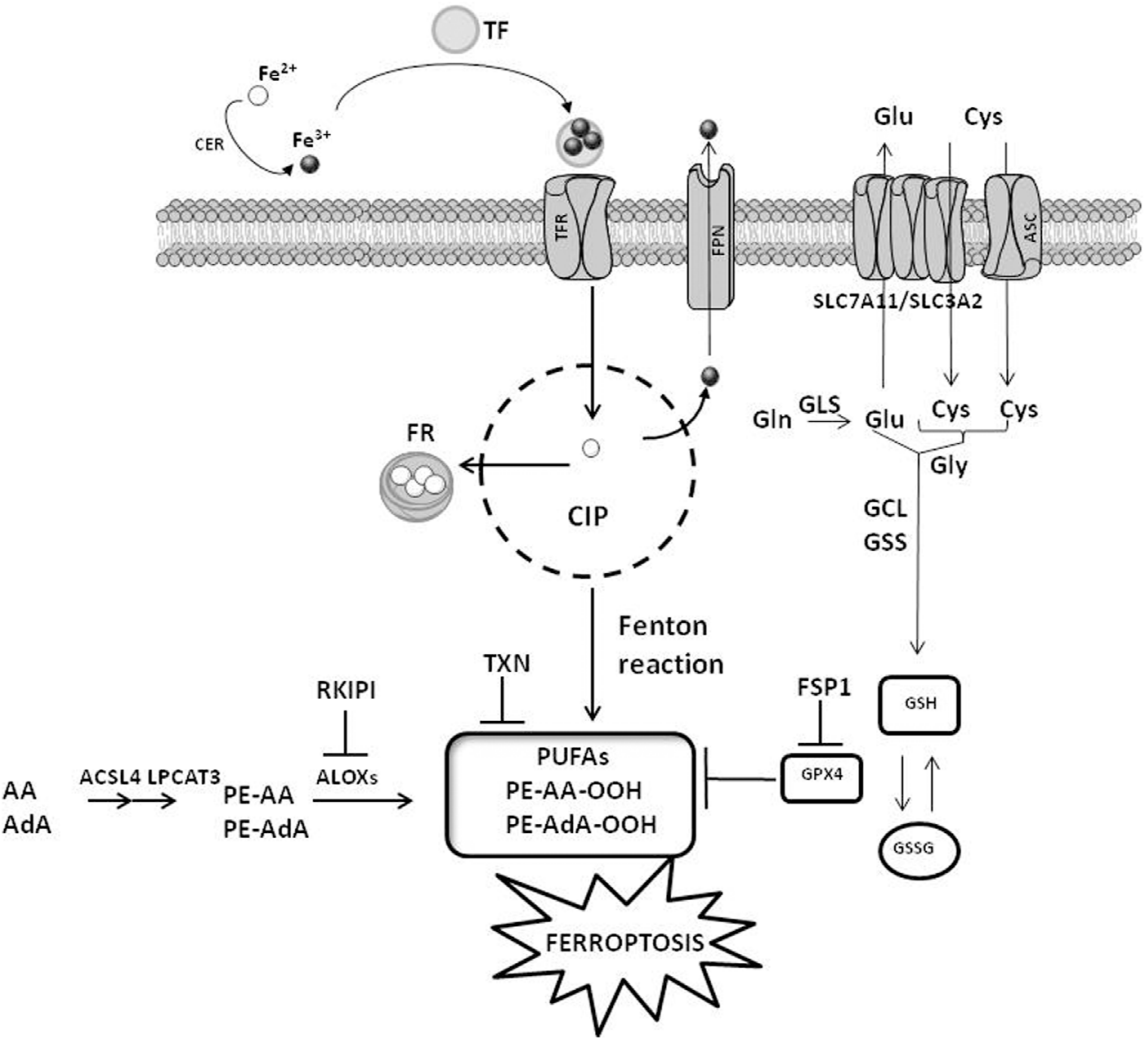
Cellular mechanisms of ferroptosis. The cellular pathways involving iron, amino acids and ROS metabolisms are reported. CIP, cellular iron pool; FR, ferritin; CER, ceruloplasmin; TF, transferrin; TFR, transferrin receptor; FPN, ferroportin; ACSL4, acyl-CoA synthetase long-chain family member 4; LPCAT3, lysophosphatidylcholine acyltransferase 3; ALOXs, arachidonate lipoxygenase; GPX4, glutathione peroxidase 4; ASC, alanine–serine–cysteine system; SLC7A11, solute carrier family 7 member 11; SLC3A2, solute carrier family 3 member 2; GLS, glutaminases; GSS, glutathione synthetase; GCL, glutamate-cysteine ligase; PUFAs, polyunsaturated fatty acids; FSP1, ferroptosis suppressor protein 1; TXN, thioredoxin. AA, arachidonoyl; AdA, adrenic acid; PE, phosphatidylethanolamines; RKIPI, Raf1 kinase inhibitory protein GSH, glutathione; GSSG, glutathione disulfide. The figure is prepared using tools from Servier Medical Art (http://www.servier.fr/servier-medical-art, accessed on March 2023).

Ferroptosis is governed by three main cellular mechanisms, including i) iron metabolism, ii) amino acid metabolism and iii) ROS metabolism (Figure 1) (Liang et al., 2019).

### 2.1 Iron metabolism

Iron transport systems regulate iron accumulation and in turn ferroptosis induction. These transporters, including ceruloplasmin (CER), transferrin (TF), transferrin receptor (TFR), ferritin (FR) and ferroportin (FPN), impact on intracellular levels of iron. Adsorbed Fe^2+^ is oxidized to Fe^3+^ by CER and in this form is captured by TF. The interaction of TF with TFR favors the cellular uptake of iron. Upon reduction to Fe^2+^ by the sixtransmembrane epithelial antigen of the prostate 3 (STEAP3), iron is bound to FR or stored into the cellular iron pool (CIP). When cells are saturated by iron, exceeding amounts of Fe^2+^ are oxidized to Fe^3+^ and pumped out of the cells by FPN (Trujillo-Alonso et al., 2019). CIP is controlled by other two factors: the nuclear receptor coactivator 4 (NCOA4), which is a specific receptor favoring FR accumulation into the autophagosome, and the iron-responsive element binding protein 2 (IREB2), which is a transcription factor controlling the iron metabolism by regulating the level of FR (Dixon et al., 2012; Mancias et al., 2014; Tang et al., 2018). Interferences with the iron balance regulated by these mechanisms (e.g., increased uptake or reduced export) stimulate iron-mediated lipid peroxidation and ferroptosis (Yang and Stockwell, 2008; Stockwell et al., 2017).

### 2.2 Amino acid metabolism

The exchange of cystine/cystathionine across the plasma membranes depends on the red-ox state of the extracellular compartment. Under reducing conditions, the intracellular accumulation of cysteine is controlled by the alanine–serine–cysteine (ASC) system. Conversely, oxidative extracellular conditions stimulate the exchange of cystine/cystathionine with glutamate mediated by Xc–transporter system (Doll and Conrad, 2017). Two subunits linked via a disulfide bridge compose the Xc–, including the catalytic subunit solute carrier family 7 member 11 (SLC7A11) and the regulatory subunit solute carrier family 3 member 2 (SLC3A2). Intracellular glutamate levels, which are under the control of the enzymatic activity degrading glutamine (glutaminolysis) mediated by glutaminases (GLS) 1 and 2, impact on Xc–activity. Diminished cellular content of cysteine, which is stimulated by enhanced GLS activity or reduced SLC7A11 levels as well as reduced activation of spermidine/spermine N1 acetyltransferase 1 (SAT1), favors lipid peroxidation and ferroptosis induction (Jiang et al., 2015; Jennis et al., 2016; Ou et al., 2016; Zhang et al., 2018). Alterations in GLS, SLC7A11 or SAT1 activities, which result in reduced intracellular availability of cysteine, negatively impact on glutathione (GSH) levels triggering ferroptosis (Shah et al., 2017). The synthesis of GSH needs glutamate, cysteine, and glycine and is catalyzed by glutamate-cysteine ligase (GCL) and glutathione synthase (GSS). The erastin-mediated inhibition of Xc–, which reduces cystine uptake, or the inhibition of GSH biosynthesis via buthionine sulfoximine, deplete intracellular GSH levels leading to ferroptosis.

### 2.3 ROS metabolism

Besides DNA damage, ROS stimulate ferroptosis by provoking alterations in lipid metabolism (D’Herde and Krysko et al., 2017; Lin et al., 2018). The lipid peroxidation occurring in PUFA is among the most important type of cellular damage for ferroptosis induction, and cells with high levels of PUFA are very sensitive to ferroptosis (Yang et al., 2016; Yuan et al., 2016). The catalytic activity of two enzymes, acyl-CoA synthetase long-chain family member 4 (ACSL4) and lysophosphatidylcholine acyltransferase 3 (LPCAT3), which allows the esterification and incorporation of PUFA into membrane phospholipids, is crucial for sensitizing cells to ferroptosis. The accumulation of lipid peroxides enhances the formation of additional ROS that increases biomacromolecule damage leading to cell membrane destabilization and micelle formation, in turn enhancing ferroptosis induction (Gaschler and Stockwell, 2017; Feng and Stockwell, 2018). Two proteins are critical for ferroptosis induction as well, the small scaffolding protein Raf1 kinase inhibitory protein (RKIP1) and the seleno enzyme glutathione peroxidase 4 (GPX4) (Wenzel et al., 2017; Tang et al., 2018; Liang et al., 2019). By interacting with the iron-containing enzyme arachidonate lipoxygenase 15 (ALOX15), RKIP1interferes with the production of phospholipid alcohols regulating ferroptosis. Similarly, GPX4 is a detoxifying enzyme catalyzing the transformation of PUFA into non-toxic phospholipid alcohols. The red-ox reaction involving the oxidation of GSH into GSSG is required by GPX4 to accomplish its catalytic activity, and cells showing high levels of GPX4 are less susceptible to ferroptosis (Seibt et al., 2019). On the contrary, compounds impairing the activity of GPX4 by reducing its expression/activity are typical ferroptosis inducers (Tang et al., 2018; Liang et al., 2019). Since the activity of GPX4 needs selenocysteine tRNA, whose cellular amount is controlled by the mevalonate (MVA) pathway, the inhibition of MVA pathway favors ferroptosis by reducing the availability of selenocysteine tRNA (Kryukov et al., 2003). Another protein controlling GPX4 activity is the ferroptosis suppressor protein 1 (FSP1). FSP1 inhibits GPX4 and its expression is high in cells resistant to ferroptosis. Elevated levels of FSP1 protect cells against compounds that induce ferroptosis by targeting GPX4 (Bersuker et al., 2019; Doll et al., 2019). Upon myristoylation, the cytoplasmic FSP1 moves to the plasma membranes and in this peculiar cellular localization catalyzes the reduction of coenzyme Q10. This behavior, in presence of GSH as well as active GPX4, attenuates the propagation of lipid peroxide production and reduces phospholipid peroxidation attenuating ferroptosis (Doll et al., 2019).

## 3 Cross-talk between radiotherapy and ferroptosis in cancer

Besides direct damage of biomacromolecules (e.g., DNA, proteins and lipids), radiotherapy generates ROS, which are the most important molecules responsible for lipid peroxide accumulation and ferroptosis. The relationship between radiotherapy and ferroptosis is corroborated by several evidence, including the specific staining (e.g., C11-BODIPY) as well as the increased expression of specific markers (e.g., MDA, 4-HNE and prostaglandin-endoperoxide synthase 2, PTGS2) reflecting lipid peroxidation observed in IR-exposed cancer cell lines and tumor samples. Moreover, irradiated cells show morphological alterations of mitochondria typical of ferroptosis (Lang et al., 2019). Of note, these features depend on IR doses administered. In support of the cross-talk between radiotherapy and ferroptosis is the observation that treatment of cells with iron chelators (e.g., deferoxamine) or with ferroptosis inhibitors (e.g., ferrostatin-1 and liproxstatin-1) before exposure to IR partially rescue their survival, and that this finding is more evident in comparison to what observed combining IR with compounds that inhibits other RCD (Lei et al., 2021).

**FIGURE 2:**
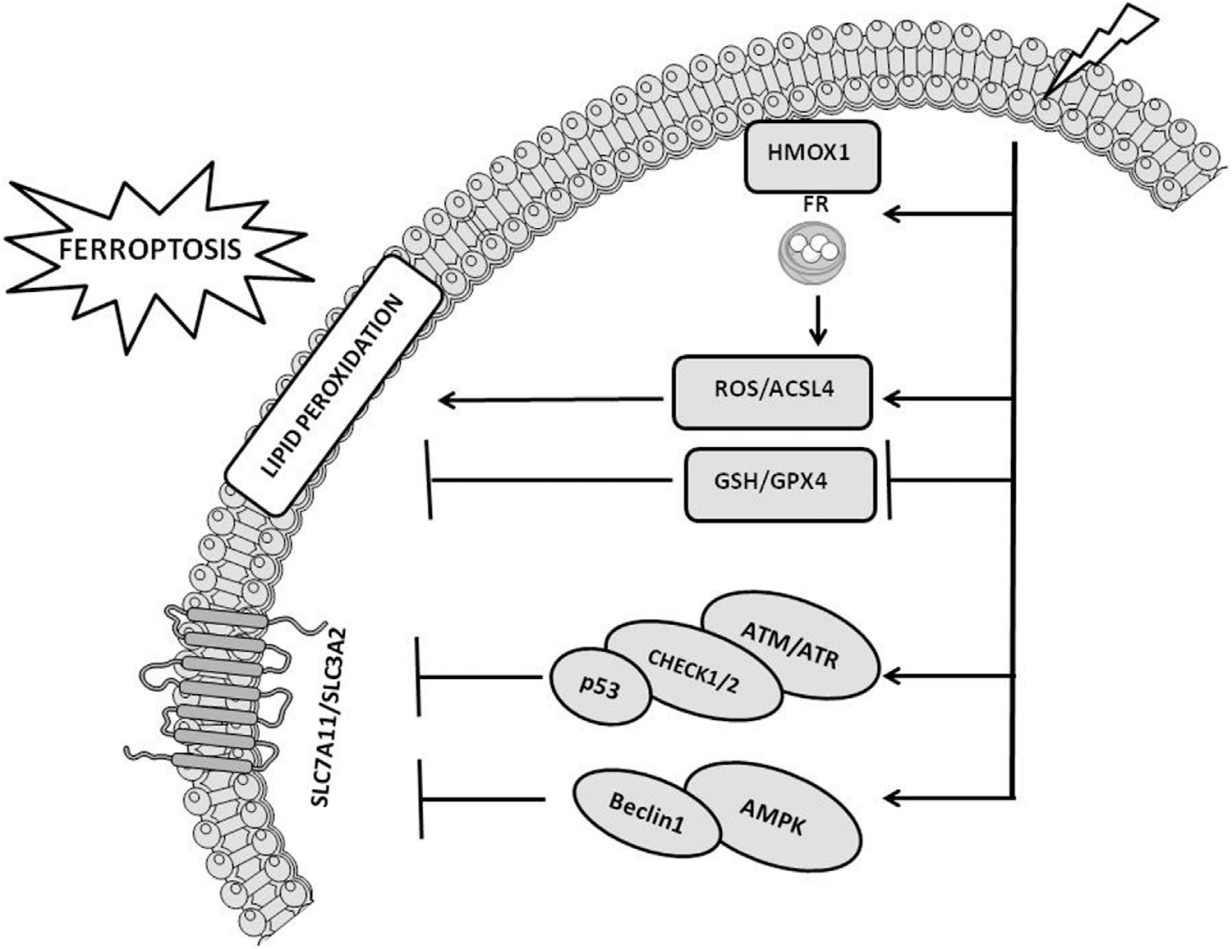
Cellular pathways implicated in radiotherapy-induced ferroptosis. The figure reports the cross-talk occurring between ionizing radiation and ferroptosis pathways, including ROS/ACSL4, GSH/GPX4, SLC7A11, ATM/ATR, and AMPK pathways. The figure is prepared using tools from Servier Medical Art (http://www.servier.fr/servier-medical-art, accessed on March 2023).

Three major pathways regulate IR-mediated ferroptosis induction (Lang et al., 2019; Lei et al., 2020; Ye et al., 2020) (Figure 2), including 1) ROS and ACSL4. Increased ROS produced by IR are responsible for the formation of PUFA radicals that, following the interaction with oxygen (Fenton reaction), generate lipid hydroperoxides (PUFA-OOH) (Lei et al., 2020). This pathway is powered by the IR-induced ACSL4 expression (Figure 1) that, together with LPCAT3, favors the synthesis of phospholipid containing PUFA, which are also liable of peroxidation; 2) GSH and GPX4. IR exposure depletes GSH leading to reduced activity/expression of GPX4 and in such a way attenuating the GPX4-mediated detoxification functions leading to increased toxic effects of lipid peroxides (Ye et al., 2020) and 3) SLC7A11. Reduced levels of SLC7A11 favor ferroptosis by downregulating cystine uptake and in turn GSH synthesis and GPX4 functions (Figure 1). Through the activation of ataxia telangiectasia mutated serine/threonine kinase (ATM), IR represses SLC7A11 levels stimulating ferroptosis. Other studies have underlined that the expression of SLC7A11 is increased upon IR exposure leading to the interpretation that an adaptive cellular response to IR rescues the SLC7A11 expression or that the level of SLC7A11 upon IR exposure depends on a peculiar cellular context (Xie et al., 2011; Lei et al., 2020).

The activation of the cellular pathways induced by the DNA damage together with the stimulation of signaling pathways associated with alterations of lipids contained into the cellular membranes occurring upon radiotherapy exposure synergize each other leading to enhanced tumor growth inhibition (Lei et al., 2020). IR-induced DNA damage increases the expression of sensor proteins, including ATM and ataxia telangiectasia and Rad3 related serine/threonine kinase (ATR) that recognize the altered DNA and stimulate DNA damage response signaling cascades (DDR). DDR activate checkpoint kinases 1/2 (chk1/2) and in turn stimulate the phosphorylation of p53 inducing cell-cycle arrest. The block of the cell-cycle is required by the cells to test the severity of the damage and “decide” their fate, 1) survive, upon the activation of the DNA repair machine in case the damage can be corrected, or 2) comit suicide, in case the damage is irreparable or not correctly repaired, triggering RCD, including ferroptosis (Maier et al., 2016; Huang and Zhou, 2020). Upon p53-mediated cell-cycle arrest, irradiated cells mostly activate senescence (Bieging et al., 2014; Maier et al., 2016). P53 mutation, which is a very common condition in tumors, engage an alternative senescence checkpoint protein, p16-retinoblastoma (RB) (Sabin and Anderson, 2011). Senescence can coexists with apoptosis and in case of prolonged p53 activation, IR preferentially stimulates both intrinsic (e.g., PUMA, BAX, NOXA, cytochrome C and caspase-9/3/7) and extrinsic apoptosis (e.g., FAS/CD95, DR5, FAS ligands and caspase-8) rather than senescence (Sheikh and Fornace, 2000; Aubrey et al., 2018; Mijit et al., 2020). Besides the above mentioned RCD, radiotherapy can induce autophagy and necroptosis. Since autophagy has both pro-survival and pro-cell death properties, controversial and not completely elucidated is its role in IR response (Hu et al., 2016). Similarly, also the role of necroptosis in radiation response is ambiguous (Adjemian et al., 2020).

ATM activated by DNA damage increases p53 expression that reduces the levels of SLC7A11 via a repressing interaction with SLC7A11 promoter or by stimulating USP7-mediated proteasome degradation of SLC7A11, in turn leading to ferroptosis (Kang et al., 2019; Wang et al., 2019). Similarly, p53 induces ferroptosis by stimulating the expression of SAT1, GLS2 or ferredoxin reductase (FDXR) (Hu et al., 2010; Ou et al., 2016; Zhang et al., 2017). In addition, p53 controls the expression of MDM2 that, via regulating lipid metabolism and FSP1 expression, favors ferroptosis (Venkatesh et al., 2020). Conversely, ferroptosis is attenuated upon p53-mediated upregulation of p21. Metabolic stress induced by cystine deprivation stimulates the p53-p21 axis preserving GSH levels and attenuating ferroptosis (Tarangelo et al., 2018). Another protein involved in IR-induced ferroptosis is the AMP-activated protein Kinase (AMPK) (Lei et al., 2020; Ye et al., 2020). This protein can either stimulate or inhibit ferroptosis. By stimulating the phosphorylation of beclin 1, which in turn favors the downregulation of Xc–, activated AMPK induces ferroptosis (Song et al., 2018). In the other hand, AMPK activates the biosynthesis of PUFA containing phospholipids inhibiting ferroptosis (Lee et al., 2020). IR stimulates the expression of heme oxygenase 1 (HMOX1) and FR, which trigger the release of iron and in such a way the induction of ferroptosis (Chiang et al., 2018). Conversely, the exposure to IR upregulates FR heavy chain (FTH1) and reduces oxidative stress in turn attenuating ferroptosis and promoting radiation resistance (Choudhary et al., 2020). These findings indicates an intersection between cellular pathways implicated in DNA damage response and RCD, which includes, besides ferroptosis, the immunogenic cell death (ICD) mechanisms embracing apoptosis, necroptosis and autophagy. Upon radiotherapy-mediated ICD stimulation, T-cells recruited into the tumor microenvironment (TME) promote ferroptosis (Lang et al., 2019).

### 3.1 Combination strategies for enhancing ionizing radiation antitumor activity and for overcoming radiation resistance

Radiation resistance of tumors is an urgent clinical problem responsible for treatment failure. The understanding of the molecular mechanisms subtending radiation resistance is critical for setting up medical strategies, including drug combinations, aimed at improving rediotherapy response (Table 1).

**TABLE 1:** Combination strategies for enhancing ferroptosis induced by ionizing radiation.

Tumor type	Cell lines	Pathway involved	*In vivo* evaluation	Combination strategy	References
Nasopharyngeal carcinoma	C666-1	FTO/OTUB1/SLC7A11	HONE1R	IR/FB23-2	Huang et al. (2023)
	HONE1				
	C666-1R				
	HONE1R				
Lung cancer	A549	ANGPL4/GPX4/SLC7A11	A549	IR/ANGPTL4 enriched exosomes	Zhang et al. (2022)
	H1299				
Hepatocellular carcinoma	SK-Hep	SOCS2/SLC7A11	SK-Hep-1 SK-Hep-1R	IR/SOCS2-overexpressing plasmid	Chen et al. (2023)
	HepG2				
	SK-Hep-1R				
	HepG2-1R				
Hepatocellular carcinoma	HepG2	COMMD10/HIF1α/SLC7A11	HepG2	IR/Cu chelator	Yang et al. (2022)
	MHCC-97H				
Oral squamous cell carcinoma	SCC15-S	GPX4	SCC15-R	IR/Hyperbaric oxygen (HBO)	Liu et al. (2022)
	SCC15-R				
Colorectal cancer	MC38	ATF3-SLC7A11-GPX4	MC38	IR/niraparib	Shen et al. (2022)
	CT26				
	HT29				
Esophageal squamous cell carcinoma	KYSE30	SCD1	N.D.	IR/MF-438	Luo et al. (2022)
	KYSE70				
	KYSE140				
	KYSE150				
	KYSE410				
	KYSE450				
	KYSE510				

N.D., not defined.

Radiotherapy is used for the clinical management of patients with different tumor types including nasopharyngeal carcinoma (NPC) and the development of radiation resistance is the main cause of treatment failure in NPC-suffering patients. Huang et al. report that these patients show increased expression of m6A mRNA demethylase fat mass and obesity-associated protein (FTO) and that this feature correlates with radiation resistance and poor prognosis (Huang et al., 2023). In support of this observation is the finding that increased levels of FTO characterize NPC cell lines resistant to IR (C666-1R, HONE1R) compared to the corresponding sensitive counterparts (C666-1, HONE1). NPC cells exposed to IR show morphological changes typical of ferroptosis, increased MDA levels and reduced GSH cellular content. The treatment with FB23-2 (a FTO inhibitor) counteracts this behavior, which is reversed following FTO overexpression or upon exposure to ferrostatin-1. Moreover, the IR response of resistant cells is significantly improved by FB23-2 treatment. The exposure to IR and FB23-2 increases DNA damage *in vitro* and this observation is corroborated *in vivo* in xenograft HNE1R-bearing mice. The authors speculate that a link exists between FTO and OTU deubiquitinase, ubiquitin aldehyde binding 1 (OTUB1). Molecularly, via its demethylase activity, FTO produces m6A modification of the OTUB1 transcript stimulating the expression of OTUB1 protein and its interaction with SLC7A11, in turn activating SLC7A11 and leading to ferroptosis inhibition and radiation resistance. These interpretations are supported *in vivo* in xenograft HONE1R-bearing mice and PDX-mouse models exposed to the combination erastin/IR. Compared to IR alone, the combination with the ferroptosis inducer erastin enhances the radiosensitivity of HONE1R tumors.

Another key protein implicated in radiation resistance by inhibiting ferroptosis is angiopoietin-like 4 (ANGPTL4) (Zhang et al., 2022). High levels of ANGPTL4 associate with poor prognosis of patients suffering from lung adenocarcinoma and adrenocortical carcinoma. A549 and H1299 lung cancer cell lines cultured under hypoxic condition show an increased expression of ANGPTL4 and radiation resistance. Moreover, compared to cells cultured under physiologic conditions, hypoxic cells show higher levels of ANGPTL4 accumulated into released exososmes. Upon silencing of ANGPTL4, A549 cells restore the sensitivity to IR, while reacquire IR resistance after ANGPTL4 ectopic expression. The relationship between ANGPTL4 and ferroptosis is demonstrated in ANGPTL4-overexpressing cells cultured under normoxia, which show increased expression of GPX4, SLC7A11, FTH1, and FTL (e.g., reduced ferroptosis and enhanced radiation resistance). Conversely, the above ferroptosis-associated proteins are decreased in ANGPTL4-silenced cells cultured under hypoxic conditions (e.g., increased ferroptosis and radiosensitivity). *In vivo* experiments in xenograft A549-bearing mice treated with ANGPTL4 enriched exosomes and exposed to IR support *in vitro* data confirming the relationship between radiation resistance and ferroptosis.

Radiation resistance accounts for the failure of radiotherapy in hepatocellular carcinoma (HCC) as well and Chen and colleagues have studied the role played by ferroptosis in radiosensitizing HCC using a panel of radiosensitive (SK-Hep and HepG2) and radioresistant (SK-Hep-1R and HepG2-1R) HCC cell lines (Chen et al., 2023). By comparing the gene expression profiles of SK-Hep and SK-Hep-1R cells, SOCS2, and SMOX genes were identified as differentially expressed. The expression of SOCS2 gene was reduced in SK-Hep-1R, while SMOX was increased. Gene expression data from GEPIA and HCCDB databases and Kaplan-Meier (K-M) analysis showed that low expression of SOCS2 and high expression of SMOX correlate with poor prognosis of HCC patients. The radiation resistance observed in resistant cells is overcome upon SOCS2 ectopic expression. Conversely, radiation resistance is enhanced in SK-Hep and HepG2 cells following SOCS2 silencing. *In vivo* experiments confirm the *in vitro* results. Moreover, immunohistochemical staining of irradiated explanted tumors shows an increased expression of SOCS2 paralleled by a modulation of ferroptosis markers, including a downregulation of GPX4 and SLC7A11, and an increased expression of 4-HNE. This observation is confirmed in a set of clinical tissues in which the levels of ferroptosis markers correlate with radiotherapy response. Co-immunoprecipitation assays demonstrate a protein interaction between SOCS2 and SLC7A11. Following the protein interaction, the E3-ubiquitin ligase activity of SCOS2 allows ubiquitination and proteasomal degradation of SLC7A11 leading to ferroptosis.

Iron homeostasis is controlled by copper (Cu) and elevated levels of this micronutrient impact on tumorigenesis, resistance to treatments and prognosis of HCC suffering patients (Yang et al., 2022). HepG2 and MHCC-97H cells exposed to IR downregulates the copper metabolism MURR1 domain 10 (COMMD10) increasing the cellular accumulation of Cu and leading to radiation resistance. These results are corroborated by gain and loss of function experiments performed in a panel of HCC cell lines showing that increased COMMD10 levels parallel with reduced cellular accumulation of Cu and radisensitivity. Conversely, reduced expression of COMMD10 increases Cu accumulation leading to radiation resistance. The role played by Cu in tumor growth is confirmed *in vivo* in HepG2-bearing mice fed with Cu-rich food upon irradiation. Compared to mice fed under normal conditions, animals fed with Cu-rich food show increased tumor volume, which is reduced following the administration of a Cu chelator. Moreover, immunohistochemistry analysis of HCC clinical samples shows that the levels of COMMD10 in patients sensitive to radiotherapy are higher than that measured in samples from radioresistant subjects. Proteomic analysis performed in HepG2 and COMMD10-depleted HepG2 cells reveals a differential expression of genes implicated in cell death. These findings, together with the observation that the treatment with ferrostatin-1 and liproxstatin-1 increases the growth of COMMD10 overexperring cells, indicate that ferroptosis is critical for radiosensitivity. Based on the observation that the sequence of the promoter of SLC7A11 contains binding sites for HIF1-α, the authors propose an interplay between COMMD10, SLC7A11, and HIF1-α as a molecular mechanism supporting the cellular response. By interacting with HIF1-α, COMMD10 impedes HIF1-α nuclear translocation and reduces SLC7A11 expression. The IR-mediated reduced expression of COMMD10 favors the cellular accumulation of Cu that stabilizes HIF1-α and inhibits its ubiquitin degradation. Following nuclear translocation, HIF1-α stimulates CER and SLC7A11 transcription inhibiting ferroptosis.

The effect of the co-exposure of IR and hyperbaric oxygen (HBO) on oral squamous cell carcinoma (OSCC) cells is studied by Liu and co-workers (Liu et al., 2022). Compared to the exposure to IR, the combination IR/HBO increases the cytotoxicity in SCC15-S cells, which is only in minimal part mediated by apoptosis. Conversely, the combination enhances the levels of ferroptosis markers (iron, ROS and MDA). Upon IR exposure, cells increase the levels of ACSL4 and SLC7A11 (two ferroptosis promoters) as well as that of GPX4 (a ferroptosis blocker), the latter being the major factor accounting for radiation resistance. The treatment with IR/HBO is ineffective on ACSL4 and SLC7A11 levels, while reduces the expression of GPX4 and in such a way shifts the equilibrium towards the induction of ferroptosis. These findings are corroborated by the observation that the transfection of SCC15-S cells with GPX4-overexpressing plasmid or the treatment with ferrostatin-1 reverse the effects of the combination. The authors also show that IR/HBO exposure sensitizes the radio-resistant SCC15-R cells to IR. Although less sensitive to ferroptosis, SCC15-R cells exposed to the combination significantly reduce the expression of GPX4 in turn favoring ferroptosis, and these results are confirmed *in vivo* in xenograft SCC15-R-bearing mice. Compared to mice treated with IR, animals exposed to IR/HBO show a significant reduction in tumor growth. In support of the ferroptosis induction as a mechanism of tumor growth delay is the observation that the treatment with ferrostatin-1 counteracts the antitumor activity of IR/HBO exposure. A deeper investigation on clinical samples (tumor and normal tissues as well as serum) from 38 OSCC patients shows that, compared to adjacent normal oral tissues, the expression of GPX4 is increased in tumors. In addition, the serum levels of GPX4 in cancer patients are increased in comparison to healthy donors. Of note, high levels of GPX4 (e.g., reduced ferroptosis) are accompanied with poor chemo-radiotherapy outcome.

Another interesting combination for sensitizing colorectal cancer (CC) to IR is proposed by Shen and colleagues (Shen et al., 2022). To protect themselves against DNA damages, cancer cells stimulate poly (ADP-ribose) polymerase (PARP)-1 and PARP-2 activities favoring the activation of DNA repair pathways. This scenario supports the rational of combining radiotherapy with PARP inhibitors (PARPi) to potentiate IR-mediated DNA damage and in turn increasing cell death. Compared to CC cell lines (murine MC38 and CT26 cells as well as human HT29 cells) exposed to IR, cells exposed to the combination of the PARPi niraparib with IR show increased levels of DNA DSBs. The combination significantly increases the cell death *in vitro* and enhances antitumor effects *in vivo*. The exposure to PARPi induces the cyclic GMP-AMP synthase (cGAS) and stimulator of interferon genes (STING), allowing the activation of cGAS-STING-TBK1-IRF3 signaling that stimulates IFNB1 transcription and the release of IFNβ, CXCL10, CCL5, and MX1. These findings are also observed *in vivo* in M38 tumor-bearing mice. The role played by cGAS is corroborated by the observation that cGAS-silenced M38 cells are less sensitive to the combination treatment. The levels of ferroptosis markers (increased MDA and PTGS2 levels, reduced SLC7A11 and GPX4 expression) reflect the induction of ferroptosis as a mechanism of cell death. The analysis of the gene expression of cGAS-silenced M38 cells in comparison to cGAS normal expressing cells supports a critical role for the activating transcription factor 3 (ATF3) and underlines the existence of a ATF3-SLC7A11-GPX4 axis controlling ferroptosis induction upon exposure to IR and niraparib. In addition, cGAS depletion in M38 tumor-bearing mice abolishes the IR-induced infiltration of CD8+T, CD8+GZMB + T-cells leading to reduced antitumor efficacy, thus corroborating the role of cGAS for the combination efficacy. The analysis of tumor samples before and after radiotherapy from 32 patients affected by CC reveals that increased expression of cGAS, ATF3, and PTGS2 as well as an high density of CD8+T-cells associate with a high disease-free survival rate.

The enzyme stearoyl-CoA desaturase (SCD1) catalyzes the formation of oleic acid and palmitoleic acid and plays a critical role in IR response. Increased levels of SCD1 are observed in a panel of esophageal squamous cell carcinoma (ESCC) cell lines and the targeting of SCD1 by MF-438 is pursued by Luo and colleagues for increasing IR potency (Luo et al., 2022). Cells treated with MF-438 reduce cell growth and the combined exposure to IR and subtoxic concentrations of MF-438 results in synergistic antiproliferative activity. The synergism is attenuated upon silencing of SCD1 as well as following exposure to the ferroptosis inhibitor RLS3. The MF-438-mediated inhibition of SCD1 increases lipid peroxidation, ATP and HMGB1 release into the extracellular compartment, and this behavior is enhanced upon exposure to the combination IR/MF-438. Since similar results are observed in cells exposed to the combination IR/RLS3, it is likely that the induction of ferroptosis is the key mechanism of antitumor activity. These observations are corroborated by the finding that cells exposed to exogenous oleic acid or palmitoleic acid undergo ferroptosis. *In vitro* data are confirmed *in vivo* in ESCC-bearing mice exposed to IR/MF-438. The authors analyze the expression of SCD1 in ESCC patients from GEPIA database and stratify them in high and low expression groups. Compared to normal epithelium, SCD1 is significantly increased in tumor tissues and high SCD1 expressing patients experience a shorter disease-free survival.

### 3.2 Nano-systems for enhancing ionizing radiation antitumor activity

Another strategy for potentiating IR-mediated ferroptosis induction implies the use of metal-based nanoparticles (NPs) (Table 2). These NPs exploit the enhanced permeability retention effect to selectively induce ferroptosis in tumors. The tumor selectivity is also enhanced by the in local IR exposure, and these features allow reduced treatment toxicity.

**TABLE 2:** Nano-systems for enhancing the antitumor activity of ionizing radiation.

Nano-system	Type of metal	Cell lines	*In vivo* evaluation	References
HPNPs	Iron	Mouse melanoma B16F10 cells	Mouse melanoma B16F10 tumors	Lin et al. (2023)
		Mouse normal L929 fibroblasts		
FPH	Iron	Mouse mammary carcinoma 4T1 cells	Mouse mammary 4T1 tumors	Hou et al. (2023)
		Mouse RAW264.7 macrophages		
Fe_2_O_3_@TA-Pt	Iron	Mouse mammary carcinoma 4T1 cells	Mouse mammary 4T1 tumors	Jiang et al. (2022)
		Normal human HUVEC cells		
GOD@FeN_4_-SAzyme	Iron	Mouse mammary carcinoma 4T1 cells	Mouse mammary 4T1 tumors	Zhu et al. (2022)
SPIONC	Iron	Human lung cancer NCI-H460 cells	Human lung cancer NCI-H460 tumors	Li et al. (2022)
iCoDMSN	Cobalt	Mouse mammary carcinoma 4T1 cells	Mouse mammary 4T1 tumors	Zhao et al. (2023)
		Human breast cancer MCF-7 cells		
		Human lung cancer A549 cells		
		Human colorectal cancer Caco-2 cells		
		Human gastric carcinoma SGC-7901 cells		
AGulX	Gadolinium	Human breast cancer MDA-MB-231 cells	Human breast cancer MDA-MB-231 tumors	Sun et al. (2022)
		Human breast cancer MDA-MB-468 cells		
3a	Gold	Human cervical carcinoma HeLa cells	Human cervical carcinoma HeLa cells transplanted in zebrafish	Yang et al. (2022)
		Human cervical carcinoma SiHa cells		
PBmB-DOX	Bismuth	Mouse mammary carcinoma 4T1 cells	Mouse mammary 4T1 tumors	Hou et al. (2022)

#### 3.2.1 Iron-based nanosystems

Lin and coworkers have assembled hemin, PX-12 (a TRX-1 inhibitor) and human serum albumin to built HPNPs (Lin et al., 2023). These NPs are stable in physiologic solution and in blood and rapidly release PX-12 under acidic conditions (e.g., pH 5). Acidic conditions, recapitulating acidic TME, favor the production of OH• by HPNPs and stimulate Fenton reaction. *In vitro* experiments performed in mouse melanoma B16F10 cells and normal mouse L929 fibroblasts show that HPNPs are better internalized in tumor compared to normal cells. The combination HPNPs/radiotherapy improves ROS production leading to increased cytotoxicity with respect to HPNPs administered alone. Besides the increased ROS production (mediated by both hemin and radiotherapy), the combination HPNPs/radiotherapy implements MDA, reduces the levels of antioxidants (GSH and TRX-1) and attenuates the GPX4 activity, in turn stimulating ferroptosis. These findings are corroborated by the observation that the treatment with ferrostatin-1 counteracts this behavior. *In vivo* experiments in B16F10 tumor-suffering mice demonstrate that HPNPs are biocompatible. Moreover, compared to animals treated with HPNPs, the tumor growth of mice exposed to the combination HPNPs/radiotherapy is significantly reduced. *Ex vivo* analysis evidences reduced GSH and GPX4 levels as well as increased MDA content in tumors exposed to the combination with respect to those treated with HPNPs alone.

To potentiate radiotherapy efficacy in breast cancer, Hou et al. propose multifunctional NPs composed by a shell of platinum decorated with hyaluronic acid (HA) encapsulating a core of Fe(III)-polydopamine (FPH) (Hou et al. 2023). FPH are stable at pH 7.4 and show photothermal properties upon 808 nm irradiation. Conversely, in acidic conditions NPs dissociate and release Fe^3+^. The red-ox reaction Fe^3+^/Fe^2+^ converts GSH into GSSG allowing GSH depletion and H_2_O_2_ hydrolysis producing O_2_ and OH• (Fenton reaction). Since the depletion of GSH is increased at 50°C, it is conceivable that the photothermal-mediated hyperthermia improves antitumor potency of FPH. An additional property of FPH is their ability to produce O_2_ by catalyzing the hydrolysis of H_2_O_2_ by Pt nanoenzyme. *In vitro* experiments carried out in mouse 4T1 breast cancer cells and in RAW264.7 macrophage cells show a preferential accumulation of FPH in cancer cells, likely dependent on the interaction of HA with CD44. In addition, compared to the treatment with FPH, near infrared radiation (NIR, 808 nm) or IR alone, the exposure to FPH/NIR and FPH/IR significantly potentiates cytotoxicity in 4T1 cells. The improved cytotoxicity observed in cells treated with the combinations correlates with increased depletion of GSH levels and increased ROS and DNA damages. This behavior is counteracted by the treatment with ferrostatin-1. *In vivo* experiments performed in 4T1 tumor-bearing mice corroborate *in vitro* results and demonstrate the biocompatibility of FPH. NIR absorbance, Pt-mediated X-ray attenuation and enhanced permeability retention-mediated tumor selectivity render FPH a very useful tool for imaging as well.

Fe_2_O_3_@TA-Pt are NPs containing a core of Fe_2_O_3_ covered by platinum and tannic acid (TA-Pt) (Jiang et al., 2022). Fe_2_O_3_@TA-Pt are stable in mouse serum, while acidic pH (5.5), mimicking TME, favors the disassembling of the TA-Pt envelop releasing Fe_2_O_3_. In presence of H_2_O_2_, which is abundant in TME, Fe^3+^ is converted in Fe^2+^ generating O_2_ and OH• (Fenton reaction). Fe_2_O_3_@TA-Pt better accumulate into 4T1 cells with respect to normal HUVEC cells, and this observation correlates with the increased cytotoxicity in tumor cells. The analysis of the DNA damage upon NPs exposure reveals that, besides the ROS-mediated DNA damage, Pt-DNA adducts, which reflect the release of the Pt by NPs, are observed. Additionally, Fe_2_O_3_@TA-Pt treatment enhances the radiotherapy sensitivity of 4T1 cells and potentiates ferroptosis induction, as demonstrated by the reduced levels of GSH and GPX4 observed upon combination exposure. *In vivo* experiments in 4T1 tumor-bearing mice show the preferential accumulation of NPs in liver and tumor. Moreover, the tumor volume of mice exposed to radiotherapy upon treatment with Fe_2_O_3_@TA-Pt is significantly reduced with respect to that of animals singly treated with NPs or radiotherapy. The combination Fe_2_O_3_@TA-Pt/IR is well tolerated with no signs of toxicity, reduces the tumor recurrence as well as the pulmonary metastasis and enhances the survival of mice.

Single-atom nanozymes (SAzymes) are enzyme-based drugs containing a single metal atom in their active sites that are interesting for anticancer therapy. Critical for the antitumor properties of these nano-systems is the presence into the TME of a specific enzymatic activity as well as H_2_O_2_. Zhu et al. (2022) have engineered a SAzyme based on FeN_4_ and glucose oxidase (GOD) (GOD@FeN_4_-SAzyme) for radio-enzymatic therapy. The elevated glucose level in tumor over the normal cells allows the production of H_2_O_2_ via GOD of the GOD@FeN_4_-SAzyme and this results in sustained production of OH• and O_2_ as well as in GSH depletion. The enzymatic cascade triggered by GOD is enhanced by IR, which favor the conversion Fe^3+^/Fe^2+^ implementing the generation of OH• and potentiating apoptosis and ferroptosis. Cytotoxic experiments performed in 4T1 cells show that the killing activity of GOD@FeN_4_-SAzyme is enhanced upon exposure to IR. Compared to the treatment with GOD@FeN_4_-SAzyme or IR separately, the combination GOD@FeN_4_-SAzyme/IR significantly increases the DNA damages (increased γ-H2AX signals) as well as ferroptosis (increased lipid peroxidation and OH•, reduced GSH and GPX4 levels accompanied by mitochondria membrane alterations). Besides ferroptosis, treated 4T1 cells show apoptosis induction (e.g., PARP and caspase 3 activation). Intravenous and intratumoral injection are used for *in vivo* administration of GOD@FeN_4_-SAzyme in 4T1-tumor bearing mice. Magnetic resonance imaging (MRI) reveals that GOD@FeN_4_-SAzyme accumulate into the tumor. Compared to the exposure to GOD@FeN_4_-SAzyme or IR, a significant reduction of tumor volume is observed in animals treated with the combination GOD@FeN_4_-SAzyme/IR. *Ex vivo* investigation recapitulates *in vitro* findings (e.g., increased γ-H2Ax and reduced GSH). GOD@FeN_4_-SAzyme are biocompatible and the combination is well tolerated as well.

The pH-sensitive supramagnetic iron oxide nano-clusters (SPIONC) are proposed by Li et al. (2022) for enhancing radiation sensitivity in lung cancer. Under acidic conditions, which recapitulate TME and intracellular compartment conditions, SPIONC decompose and generate OH• via Fenton reaction. SPIONC efficiently accumulate in NCI-H460 cells. Compared to SPIONC or IR individual exposure, the combination SPIONC/IR is more effective in inhibiting cell proliferation. Cells exposed to the combination increase iron, lipid peroxides, ROS and γ-H2AX levels. These features reflect apoptosis (reduced expression of Bcl2 and increased caspase 3 activation) and ferroptosis induction (reduced expression of SLC7A11 and GPX4). The involvement of ferroptosis in SPIONC/IR response of tumor cells is corroborated by the observation that the pre-treatment with ferrostatin-1 attenuates cytotoxicity. *In vivo* experiments in orthotopic mice model of NCI-H460 cells in which SPIONC are injected by both intravenous and intra-tracheal delivery show that intra-tracheal delivery is to prefer for MRI analysis. Moreover, the exposure to the combination SPIONC/IR results in increased tumor volume inhibition and survival rate with respect to the administration of SPIONC or IR separately. No important toxic side effects are reported upon treatment. The analysis of explanted tumors from euthanized mice confirms the molecular alterations observed *in vitro*.

#### 3.2.2 Nano-systems based on cobalt, gadolinium, gold, and bismuth

Another ferroptosis-stimulating nano-system has been recently proposed by Zhao and colleagues (Zhao et al., 2023). Starting from the observation that high level of cobalt (Co) in tumors associates with a good prognosis, the authors have engineered Co oxide nanodots by assembling bovine serum albumin and CoCl_2_. These nanodots are conjugated with iRGD peptides and encapsulated into dentritic mesoporus silica nanoparticles (iCoDMSN). Acidic conditions stimulate the release of Co^2+^ by iCoDMSN and the presence of iRGD favors their tumor penetration. iCoDMSN are cytotoxic on a panel of different tumor cell lines, including murine 4T1 cells and human MCF-7 breast cancer cells, human A549 lung cancer cells, human Caco-2 colorectal cancer cells and human SGC-7901gastric carcinoma cells. Therefore, these nanostructures show interesting photoacoustic imaging ability under 808 nm irradiation and *in vivo* experiments in 4T1-tumor bearing mice show that iCoDMSN are well tolerated (e.g., no important signs of liver and kidney toxicity), preferentially co-localize with lysosomes and that iRGD favors tumor accumulation. Proteomic studies performed on 4T1 cells exposed to iCoDMSN underline that ferroptosis pathways play a critical role for cell response. These findings are supported by the increased lipid peroxidation, MDA and iron levels observed in iCoDMSN-treated 4T1 cells, and by the observation that this behavior is counteracted upon exposure to ferrostatin-1. Proteomic analysis also underlines an increased level of HMOX1, which controls Fe^2+^ accumulation and ferroptosis induction by increasing the expression of TFR as well as by reducing solute carrier family 40 member 1 (SLC40A1). Upon treatment, accumulated iCoDMSN perturb the KEAP1/NRF2/HMOX1 axis. This axis is governed by the level of nuclear factor erythroid 2-related factor 2 (NRF2, Bellezza et al., 2018), which is a transcription factor for HMOX1. These results are confirmed *in vivo* in 4T1-tumor bearing mice exposed to the combination iCoDMSN/radiotherapy. Compared to mice treated with iCoDMSN, a increased tumor volume inhibition and survival rate are observed in mice treated with iCoDMSN/radiotherapy. *Ex vivo* analysis confirms the results obtained *in vitro* (reduced KEAP1, increased NRF2, HMOX1, iron, and MDA).

Aimed at overcoming radiation resistance and reducing radiotherapy damages to normal tissues, Sun and colleagues have proposed gadolinium (Gd)-based NPs (AGulX) (Sun et al., 2022). AGulX are based on polysiloxane covering Gd entrapped by the chelator dodecane tetraacetic acid (DOTA) moieties that functionalize the polysiloxane. Upon IR exposure, AGulX produce secondary and Auger electrons as well as free radicals. These NPs are under clinical investigation in brain, lung and pancreatic cancers. In the study by Sun et al., AGulX are evaluated in triple negative breast cancer cells (MDA-MB-231 and MDA-MB-468 cell lines) *in vitro* and *in vivo*. Compared to the treatment with AGulX or IR, the combination AGulX/IR reduces cell growth as well as cell migration and invasion capability. Additionally, cells exposed to the combination enhance ROS production, DNA damage (increased number of γ-H2AX foci) as well as G2/M cell-cycle arrest. Molecularly, the combination stimulates the phosphorylation of ATR and Chk1 (e.g., G2/M block) and reduces the phosphorylation of ATM and Chk2 (e.g., reduced homologous recombination repair ability). Moreover, by diminishing the activation of the MRN-ATM-Chk2 axis, the combination also impairs the non homologous end-joining, which is reflected by the reduced phosphorylation of p53 and BRCA1. Besides apoptosis induction (e.g., PARP and caspase 3 activation), cells exposed to AGulX/IR reduce the expression of NRF2 favoring ferroptosis by attenuating SLC7A11 activity, in turn reducing GSH synthesis and GPX4 activity. The increased levels of lipid perixidation and MDA support the induction of ferroptosis as a mechanism of cell death. These findings are confirmed by the observation that ferroptosis is attenuated by the siRNA-mediated silencing of NRF2, SLC7A11 and GPX4 as well as by the treatment with ferrostatin-1. The *in vitro* results parallel the *in vivo* observations in MDA-MB-231 tumor-bearing mice. No important signs of toxicity are reported in treated animals.

A series of metal-biotin-conjugated nano-structures based on different metals endowed with radiosensitizer properties is proposed by Yang and colleagues (Yang et al., 2022). Among the different nano-systems, the gold derivative 3a is selected for further investigations. 3a contains a biotin moiety for favoring tumor selectivity and uptake, a triply bonded dicarbon alkynyl amide linker joining biotin to Au, which is hidden by a lipophilic phosphine residue to increase membrane solubility. Compared to the auranofin (the reference), 3a shows similar antiproliferative potency on human cervical carcinoma HeLa and SiHa cells. Moreover, the tumor selectivity of 3a, which is dependent on biotin, is supported by the observation that 3a uptake is higher in Hela cells (expressing high levels of biotin receptor) with respect to normal human cervical epithelial H8 cells (expressing low levels of the receptor). Au-containing compounds, including auranofin, inhibit thioredoxin reductases (TRXR) and 3a-treated cells show reduced TRXR enzymatic activity. This finding is corroborated by docking studies showing that Au binds the selenium of the TRXR. The inhibition of the detoxification properties of TRXR stimulates ROS production and favors G2/M cell-cycle arrest as well as apoptosis (e.g., reduced Bcl2, increased Bax and alteration of the mitochondria membrane potential). Gene expression analysis performed on HeLa cells exposed to 3a shows a differential expression of genes involved in ferroptosis, including TXNRD1, HMOX1, SLC7A11, GCLM, FTH1, FTL, GPX1, GPX1P1, and GPX4. The exposure of HeLa cells to IR following 3a treatment stimulates DNA damage (increased γ-H2AX levels) and downregulates GPX4 expression, leading to reduced cell survival. This behavior is counteracted by the exposure to N-acetyl L-cysteine or ferrostatin-1. The radiosensitizing properties of 3a are confirmed *in vivo* in zebrafish transplanted with Hela cells exposed to the combination 3a/IR.

Along this way, Hou et al. have reported the synthesis and the antitumor properties of PBmB-DOX NPs (Hou et al., 2022). PBmB-DOX include a core of Bi_2_S_3_ covered by PEGylated doxorubicin (DOX). Moreover, PBmB-DOX contain-Mn-O- bonds that are sensitive to high GSH level, which is typical of the TME, allowing tumor selectivity and release of DOX under acidic conditions. The PBmB-DOX disassembling mediated by high levels of GSH favors the release of Mn^2+^ that, besides stimulating Fenton reaction and potentiating DOX-mediated antitumor activity, allows magnetic resonance contrast enhancement. The downregulation of GSH reduces 4T1 cell proliferation, while no important changes are evidenced in the growth of normal PBmB-DOX-treated HUVECs cells. The depletion of GSH upon treatment reduces the expression of GPX4 and increases lipid peroxidation. PBmB-DOX are more potent than free DOX on 4T1 cells and, compared to the exposure to IR or PBmB-DOX as single treatments, the combination PBmB-DOX/IR increases the amount of γ-H2AX foci. *In vivo* studies in 4T1 tumor-bearing mice demonstrate that PBmB-DOX are biocompatible with no important toxic effects reported for major organs, and that they preferentially accumulate into the tumor. Longer circulation time in plasma is reported for PBmB-DOX with respect to free DOX, and PBmB-DOX more efficiently suppress tumor growth with respect to free DOX. The exposure to IR upon PBmB-DOX treatment significantly improves antitumor activity with no important signs of toxicity. Histological analysis of tumors explanted from mice shows increased expression of γ-H2AX and reduced GPX4 levels following the exposure to the combination. Lastly, a remarkable signal enhancement in tumor is evidenced in MRI after 6 h post-injection of PBmB-DOX, thus confirming the theranostic properties of the nano-system.

## 4 Ionizing radiation-associated gene signatures

Recently, results from investigations focused on the studies of ferroptosis-associated gene signatures for predicting radiotherapy patient outcomes have been reported (Table 3).

**TABLE 3:** Gene signatures associated to ionizing radiation.

Gene signature	Tumor types	Cell line validation	Pathway involved	References
MAPK1	Malignant glioblastoma	U87	IL-17	Xie et al. (2022)
ZEB1		U251	Cytokine-cytokine receptor interaction	
MAP1LC3A			TNF signaling pathways	
HSPB1			DCs	
CA9			Macrophages	
STAT3			TIL	
TNFAIP3			Treg cells	
SCL7A11	Breast cancer	MCF7	SLC7A11/ESR1	Liu et al. (2022)
		ZR-75-1	SLC7A11/NEDD4L	
		MDA-MB-231		
ACSL3	Prostate cancer	No	Epithelial–mesenchymal transition	Feng et al. (2022)
EPAS1			Allograft rejection	
FASN			Fc gamma R-mediated phagocytosis	
GSTP1			TGF beta signaling	
LDHB			ECM receptor interaction	
NEDD4L			Adipocytokine signaling	
			Androgen response	
			Notch signalling	

The combination of temozolomide and radiotherapy is used for the treatment of malignant glioblastoma (GBM). Though effective, the combination is not curative and the identification of radiosensitive-associated biomarkers is an urgent need for predicting prognosis and therapy outcome. In the study by Xie et al. (2022), expression profiles of genes involved in radiation response and ferroptosis-associated pathways of GBM patients and healthy subjects from The Cancer Genome Atlas (TCGA) database are analyzed. Among the differentially expressed genes (DEGs) intersecting the two pathways, seven genes (MAPK1, ZEB1, MAP1LC3A, HSPB1, CA9, STAT3, and TNFAIP3) overlap and the analysis of the protein-protein interaction network indicates STAT3 as the hub gene. The application of the Least Absolute Shrinkage and Selection Operator (LASSO) and Cox regression analysis defines a risk score that stratifies patients in low- and high-risk groups. Patients in the high-risk group show low overall survival (OS) and high mortality. Receiver Operating Characteristic (ROC) curve and K-M analysis confirm the power of the signature in predicting patients’ survival. The prognostic model is validated by data from Chinese Glioma Genome Atlas (CGGA) database used as an external independent validation cohort. Functional enrichment analyses defined by Gene Ontology (GO) and Kyoto Encyclopedia of Genes and Genomes (KEGG) as well as immune cell infiltration patterns analysis from single-sample Gene Set Enrichment Analysis (ssGSEA) allow the identification of the most represented pathways in high-risk group, including IL-17, cytokine-cytokine receptor interaction, TNF signaling pathways, DCs, macrophages, Tumor-infiltrating lymphocytes (TIL) and Treg cells. *In vitro* experiments performed in glioblastoma U87 and U251 cells treated with the combination erastin/IR support the relationship between radiosensitivity and ferroptosis.

Gene expression profiles of breast cancer and normal tissues as well as the survival and clinical information from TCGA database are analyzed by Liu and colleagues (Liu et al., 2022). Among the DEGs associated with ferroptosis, SLC7A11 is the most upregulated in tumors compared to normal tissues. Numerous clinic-pathologic properties associate with SLC7A11 levels, including the expression of estrogen receptor (ER). ER-positive tissues show lower levels of SLC7A11 (e.g., increased ferroptosis) with respect to ER-negative samples. The univariate Cox regression for OS model demonstrates that high SLC7A11 levels associate with worse OS. *In vitro* experiments carried out in a panel of breast cancer cell lines (ER-positive MCF7 and ZR-75-1 as well as ER-negative MDA-MB-231) treated with ferrostatin-1 or erastin in combination with IR support the critical role played by SLC7A11 in regulating IR-induced ferroptosis in ER-positive cells. The study also shows a positive correlation between the expression of estrogen receptor 1 (ESR1) and SLC7A11 and the analysis by K-M predicts poor prognosis for patients with high levels of ESR1. Molecularly, IR exposure stimulates the expression of ESR1 that, in turn, increases SLC7A11 levels attenuating ferroptosis. This finding is supported by the observation that upon ESR1/SLC7A11 knockdown in ER-positive cells, IR-induced ferroptosis is enhanced. Immunoprecipitation assay revels that no direct protein-protein interaction occurs between ESR1 and SLC7A11. Conversely, a protein interaction involving SLC7A11 and the E3 ubiquitin ligase neural precursor cell expressed developmentally downregulated gene 4-like (NEDD4L) is critical for stimulating the proteasome-mediated degradation of SLC7A11. Based on these findings, the authors suggest that two pathways, including ESR1/SLC7A11 and SLC7A11/NEDD4L, control SLC7A11 level and regulate ferroptosis induced by IR exposure.

By analyzing expression data (mRNA and lncRNA) from Gene Expression Omnibus (GEO) database of normal and prostate cancer tissues of patients treated with radical radiotherapy and intersecting them with ferroptosis-related genes, Feng and co-workers construct a gene signature, including ACSL3, EPAS1, FASN, GSTP1, LDHB, and NEDD4L. This signature allows the definition of a ferroptosis-related gene prognostic index (FGPI) useful for predicting biochemical recurrence (BR) and radiation resistance of prostate cancer suffering patients (Feng et al., 2022). FGPI allows the stratification of the patients in high- and low-risk groups. Although ROC curve poorly discriminates BR patients from patients who do not experience BR, it evidences that FGPI potentially reflects radiation resistance. Indeed, compared to no BR patients, a significant higher FGPI is observed for BR patients treated with radical radiotherapy. The application of the K-M curve shows that FGPI is an independent risk factor for biochemically relapse (BCR) and metastasis-free survival (MFS) in patients treated with radical radiotherapy. Moreover, compared to low-risk, high-risk group patients treated with radical prostatectomy are at higher risk of metastasis. The application of the GeneMANIA database and ceRNA network assigns a critical role to lnRNAPART1 in controlling ACSL3 and EPAS1 expression via a intricate crosstalk involving 60 different miRNAs. Gene Set Enrichment Analysis (GSEA) shows differences in pathway enrichment in high-risk (epithelial–mesenchymal transition, allograft rejection, Fc gamma R-mediated phagocytosis, TGF beta signaling pathway, and extracellular matrix receptor interaction) with respect to low-risk patients (adipocytokine signaling pathway, androgen response and notch signalling). Drug and immunologic analysis as well as TME analysis resulting from the application of dedicated softwares, which consider the expression of ACSL3 and EPAS1, underline potential sensitivity to nine drugs (OSI-027, OSI-930, PAC-1, PHA-793887, PI-103, PIK-93, SNX-2112, TPCA-1, and UNC0638) for high-risk patients. Compared to no BR patients, BR patients group shows lower expression levels of METTL14, which predict sensitivity to methylating agents, and higher expression of PDCD1LG2 (PD-L2) and CD96. However, only CD96 is significantly associated with BCR-free survival. Regarding the results of TME analysis, cancer-related fibroblasts, macrophages, stromal score, immune score, estimate score, and tumor purity are risk factors for BCR closely associated to BCR-free survival.

## 5 Conclusion

Although radiotherapy is the first choice for the treatment of different tumors types, the development of radiation resistance impairs its effectiveness and medical strategies aimed at overcoming this drawback are urgent. Among these strategies, the combination of IR with ferrptosis inducers proved to synergize thus potentiating radiotherapy. Moreover, since the activation of defense cellular pathways in response to IR exposure attenuates radiotherapy effectiveness, deeper investigations aimed at studying the involved pathways as well as at developing ferroptosis inducers or novel combinatorial strategies are intriguing ways to pave for combating radiation resistance. Besides the use of small molecules inducing ferroptosis, the combination of IR with nano-systems endowed with both diagnostic and therapeutic potential is promising. In spite of their capability to overcome the resistance to apoptosis developed by tumors exposed to conventional chemotherapeutics, ferroptosis inducers show drawbacks typical of small molecules, including low solubility, limited tumor targeting, and toxic side effects that have often impeded their clinical evaluation. These drawbacks have been tackled by NPs that, functioning like a “Trojan horse”, localize into the tumors via the enhanced permeability retention effect and are entered into the therapeutics armamentarium for fighting tumors. NPs ameliorate the circulation time of encapsulated drugs and stimulate anticancer immunity at the tumor site. Specific decoration aimed at targeting peculiar tumor-expressing molecules as well as exploiting non physiologic conditions typical of TME potentiate the tumor selectivity of NPs. Tumor targeting is also in part guaranteed by the local irradiation of the tumor. NPs are designed to disassemble themselves under peculiar conditions (e.g., the acidic pH as well as the presence of specific enzymes), ameliorating the selectivity of the cargo release. Therefore, the magnetic properties of the metal composing the structure of the NPs account for their theranostics potential. Among the combinations including small molecules here reported, only IR/niraparib is currently under investigation in phase I-II clinical trials (https://www.clinicaltrials.gov) in triple negative breast cancer (NCT03945721 and NCT04837209), pancreatic tumors (NCT04409002), prostate cancer (NCT04194554, NCT04037254), glioblastoma (NCT05666349) and head and neck squamous cell carcinoma (NCT05784012) patients. Regarding the clinical studies exploiting NPs, although all the NPs considered have been tested *in vivo* showing interesting antitumor profile, only AGluX is under phase I-II clinical evaluation in patients with brain tumors and brain metastasis, gynecologic cancers, non small cell lung cancers, and pancreatic cancers (NCT04899908, NCT03308604, NCT02820454, NCT04789486, NCT03818386, and NCT04784221. https://www.clinicaltrials.gov). Lastly, the continuous implementation of the gene signatures predicting IR response via ferroptosis-mediated cell death is expected to positively impact on patient`s health.

In conclusion, despite the preclinical success achieved with the combination strategies, additional efforts and clinical investigations are required in the future to demonstrate their safety profile as well as their antitumor effectiveness.
